# Simultaneous Down-Regulation of Dominant Cinnamoyl CoA Reductase and Cinnamyl Alcohol Dehydrogenase Dramatically Altered Lignin Content in Mulberry

**DOI:** 10.3390/plants13243512

**Published:** 2024-12-16

**Authors:** Shuai Huang, Xiaoru Kang, Rumeng Fu, Longyan Zheng, Peijun Li, Fengjuan Tang, Nan Chao, Li Liu

**Affiliations:** 1 Jiangsu Key Laboratory of Sericultural Biology and Biotechnology, School of Biotechnology, Jiangsu University of Science and Technology, Zhenjiang 212100, China; 2 Key Laboratory of Silkworm and Mulberry Genetic Improvement, Ministry of Agriculture and Rural Affairs, Sericultural Research Institute, Chinese Academy of Agricultural Sciences, Zhenjiang 212100, China

**Keywords:** cinnamyl alcohol dehydrogenase, cinnamoyl CoA reductase, *Morus*, lignin, enzymatic assay, VIGS

## Abstract

Mulberry (*Morus alba* L.) is a significant economic tree species in China. The lignin component serves as a critical limiting factor that impacts both the forage quality and the conversion efficiency of mulberry biomass into biofuel. Cinnamoyl CoA reductase (CCR; EC 1.21.1.44) and cinnamyl alcohol dehydrogenase (CAD; EC 1.1.1.95) are the key enzymes that catalyze the final two reductive steps in the biosynthesis of monolignols. In this study, we conducted a comprehensive functional analysis to validate the predominant *CCR* genes involved in monolignol biosynthesis. In this study, we initially validated the predominant *CCR* genes implicated in monolignol biosynthesis through an extensive functional analysis. Phylogenetic analysis, tissue-specific expression profiling and enzymatic assays indicated that *MaCCR1* is the authentic CCR involved in lignin biosynthesis. Furthermore, the expression level of *MaCCR1* exhibited a significant positive correlation with lignin content, and the down-regulation of *MaCCR1* via virus-induced gene silencing resulted in altered lignin content in mulberry. The down-regulation of *MaCCR1* and *MaCAD3/4,* both individually and concurrently, exhibited markedly different effects on lignin content and mulberry growth. Specifically, the simultaneous down-regulation of *MaCCR1* and *MaCAD3/4* significantly altered lignin content in mulberry, resulting in dwarfism of the plants. Conversely, the down-regulation of *MaCAD3/4* alone not only decreased lignin content but also led to an increase in biomass. These findings offer compelling evidence elucidating the roles of *MaCCRs* in mulberry and identify specific target genes, thereby providing a crucial foundation for the genetic modification of lignin biosynthesis.

## 1. Introduction

Mulberry (*Morus* spp.) is a significant economic plant in China. In addition to serving as the foundation of the traditional sericulture industry, mulberry is increasingly recognized for its potential as supplementary animal feed and as a source of bioenergy. This has attracted significant attention within the fields of animal husbandry and bioenergy production [1,2,3]. Lignin constitutes a significant component of the secondary cell wall in vascular plants [4,5], where it forms cross-links with cellulose and hemicellulose, thereby providing structural support and enhancing resistance to pathogenic invasion [4,6]. Nevertheless, the inherent recalcitrance and structural complexity of lignin pose challenges to the nutritional quality of plant feed and impede the efficiency of biomass conversion into biofuels [6,7,8]. Consequently, investigating the lignin biosynthesis pathway is of considerable importance for the targeted regulation of lignin composition and content in plants.

Cinnamoyl CoA reductase (CCR; EC 1.21.1.44) and cinnamyl alcohol dehydrogenase (CAD; EC 1.1.1.95) serve as structural enzymes facilitating the final two reductive stages of monolignol biosynthesis [9]. CCR represents the initially committed enzyme within the lignin biosynthesis pathway and is classified under the short-chain dehydrogenase/reductase (SDR) superfamily [10,11]. It can facilitate the conversion of various hydroxyl cinnamyl-CoA substrates, including *p*-coumaryl CoA, sinapyl CoA, caffeoyl CoA and feruloyl CoA, into their corresponding aldehydes: *p*-coumaraldehyde, sinapylaldehyde, caffeic aldehyde and coniferaldehyde. Subsequently, CAD utilizes these aldehydes to synthesize the corresponding alcohols [12]. Both CCR and CAD are believed to contribute significantly to the diversity of monolignols in plants and are regarded as key genes in the regulation of lignin biosynthesis [13,14].

Given the important roles of CCR and CAD in monolignol biosynthesis, genetic modification of CCR and CAD has been performed in various plant species. In summary, the regulation of *CCR* or *CAD* can significantly influence lignin content and composition, thereby impacting saccharification and cell wall digestibility [15]. Various *CCR* knock-out mutants, such as the *irx4* mutant, as well as *ccr1s* and *ccr1g,* demonstrate approximately 25–50% reductions in lignin content [16,17]. Comparable modifications in lignin content have been observed in transgenic *Arabidopsis* with down-regulated *CCR1* expression [18]. Furthermore, the down-regulation of *CCR* in poplar has been shown to decrease lignin content by 5–24%, depending on the degree of down-regulation [19]. Severe down-regulation of *CCR* in tobacco led to a significant reduction in lignin content and resulted in dwarfism in the plant [20]. In *Medicago truncatula*, the *CCR1* mutant exhibited a 50% decrease in lignin content and a reduction in the S/G ratio, whereas the *CCR2* mutant showed only a moderate decrease in lignin content but an increased S/G ratio [21]. Similar effects on lignin content and composition have been reported through the transcriptional regulation of *CAD* [13]. Specifically, *CAD* down-regulation in switchgrass resulted in a 14–22% reduction in lignin content, a decreased S/G ratio and enhanced digestibility and saccharification efficiency [22]. The *Zea mays ZmCAD2* mutant exhibited an 18% reduction in lignin content, whereas the down-regulation of *CAD* through RNA interference led to a minor decrease in the S/G ratio without affecting lignin content in maize stems [23,24]. Notably, *CAD*-RNAi maize generated increased dry biomass while maintaining a wild-type phenotype [23]. In contrast, in *Arabidopsis*, only the double mutants *cad-c, cad-d* demonstrated a reduction in lignin content, whereas single mutants *Atcad-c* or *Atcad-d* showed negligible or slight changes in lignin content [25,26]. The regulation of *CCR* or *CAD* has yielded varying outcomes depending on the plant species, the extent of transcriptional regulation and the specific *CCR* or *CAD* homologs targeted. Concurrent transcriptional regulation of *CCR* and *CAD* has also been explored as a strategy to alter lignin content and composition. In *Arabidopsis*, simultaneous suppression of *CCR* and *CAD* results in a 50% reduction in lignin content, which is associated with a pronounced dwarf phenotype and red coloration of the xylem and the similar reduction in lignin content was also reported in tobacco [27,28].

Research has indicated that both *CCR* and *CAD* are present as gene families across various plant species, including *Arabidopsis thaliana* (At), *Populus trichocarpa* (Ptr), *Eucalyptus grandis* (Eg) and *Oryza sativa* (Os) [27,29,30,31,32,33]. Our prior investigations have also identified *CCR* and *CAD* homologs in mulberry, highlighting their potential functional divergence [2]. Based on our prior research, a substantial decrease in lignin content in mulberry can be accomplished by down-regulating the expression levels of *MaCAD3/4* [34]. Consequently, it is of interest to investigate the impact on lignin content and plant growth by simultaneously down-regulating lignin-related *CCR* and *CAD* genes in mulberry. To examine the synergistic effects of *CCR* and *CAD* on lignin modification, it is essential to first identify the predominant *CCR* involved in monolignol biosynthesis in mulberry.

In our prior investigation, a total of six *CCR* and *CCR-like* genes were identified through comprehensive genome-wide screening and transcriptomic analysis [2]. In this study, four homologous genes of cinnamoyl-CoA reductase (CCR) were successfully cloned and isolated from mulberry. A comprehensive functional analysis of the CCR gene family members was conducted through tissue-specific expression profiling and *in vitro* enzyme activity assays. Additionally, the down-regulation of *CCR* homologs was employed to identify the predominant *CCR* gene involved in monolignol biosynthesis in mulberry. The concurrent down-regulation of the predominant *CCR* and *CAD3/4* genes was achieved through virus-induced gene silencing (VIGS). This simultaneous reduction in *CCR* and *CAD* expression levels resulted in a significant decrease in both lignin content and plant biomass in mulberry.

## 2. Results

### 2.1. Cloning and Characterization of CCR Homologs in Mulberry

**Figure 1:**
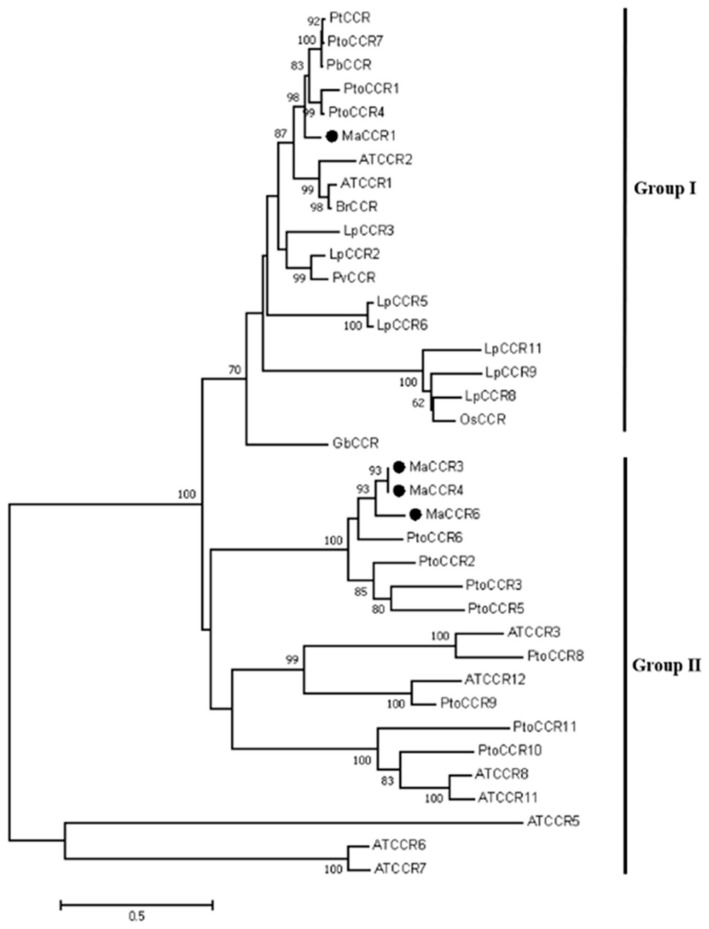
Phylogenetic analysis of CCRs in different plants. Group I comprises genuine CCRs. *MaCCRs* are marked with full black circles. Sequence information is available in Table S3.

**Figure 2:**
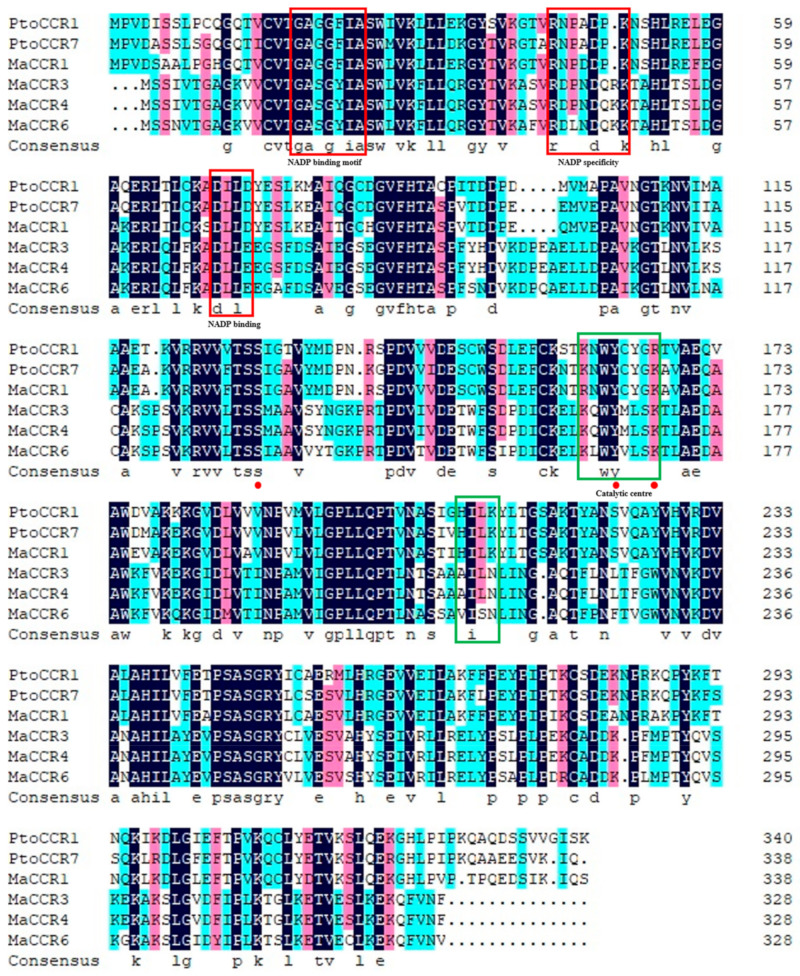
Alignment of *MaCCRs* and PtoCCR1 and 7. *Populus tomentosa* (Pto)CCR1 and PtoCCR7, which have been reported as genuine CCRs involved in lignin biosynthesis, were selected as reference CCRs. The red boxes indicate NAD(P)-binding and NADP specificity motifs, and the green boxes indicate CCR signature and substrate binding motifs (SBM). The red dots represent the catalytic triad.

Four *CCR* genes, named *MaCCR1*, *MaCCR3*, *MaCCR4* and *MaCCR6*, were successfully cloned from Morus alba var. Fengchi, while *MaCCR2* and *MaCCR5* were not obtained (Table S1). The CDS sequences of the obtained *MaCCRs* ranged in length from 987 to 1017 bp and encoded 328 to 338 amino acids, with 81.98% sequence identity among the four *MaCCRs* (Table S1). *MaCCR3* and *MaCCR4* have extremely high sequence identity, with only three different bases leading to synonymous mutations. Since *MaCCR3* and *MaCCR4* encode the same amino acid sequence, *MaCCR3/4* was used to represent *MaCCR3* and *MaCCR4* in subsequent studies. We then investigated possible functional differences among the MaCRRs. Our phylogenetic analysis revealed that *MaCCR1* was clustered with validated genuine CCRs, including *Populus tomentosa* (Pto)CCR1, 7, AtCCR, *Lolium perenne* (Lp)CCR2 and *Panicum virgatum* (Pv) CCR. These genuine CCRs have been reported to show CCR catalytic activity and were the dominant CCRs involved in lignin biosynthesis [35,36,37,38,39]. However, the other *MaCCRs* clustered in other groups, which are considered CCR-like (Figure 1). The alignment of putative MaCCR protein sequences with genuine PtoCCRs showed that four *MaCCRs* had the recognition domain of short-chain dehydrogenase (SDR), such as NAD(P)-binding and NADP specificity motifs indicated by red boxes and the conserved catalytic triad Ser-Tyr-Lys indicated by full red circles (Figure 2) [37,40]. However, in terms of the previous reported CCR signature motif and our reported CCR substrate binding motif (CCR-SBM) [40], the divergence among *MaCCRs* is obvious (Figure 2). The signature motif NWYCY located in the first green box is conserved in *MaCCR1* and PtoCCR1 and 7 while being changed in other *MaCCRs*. The same situation was also observed for CCR-SBM HXXK (green box 2), which is also conserved in *MaCCR1* and PtoCCR1 and 7, differing from other *MaCCRs* (Figure 2). These results preliminarily indicate that *MaCCR1* is the bona fide CCR in mulberry.

### 2.2. Expression of MaCCR1 in the Xylem Suggests a Possible Role in Lignification in Mulberry

**Figure 3:**
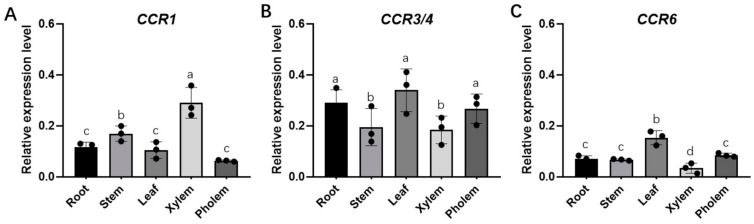
Expression profiles of several *MaCCRs*. (**A**) Quantitative real-time PCR analysis of *MaCCR1*, (**B**) *MaCCR3/4* and (**C**) *MaCCR6* in different tissues. Data are presented as means ± SD of three biological replicates. One-way ANOVA and Tukey’s multiple comparisons test were performed. Different letters indicate significant differences (*p* < 0.05).

The expression patterns of *MaCCRs* in different tissues of *Morus alba* varied considerably. Due to the extremely high sequence identity of *MaCCR3* and *MaCCR4*, the common qRT-PCR primers were used in this study to explore the expression levels of *MaCCR3* and *MaCCR4*, which were described as *MaCCR3/4*. *MaCCR1*, which was primarily considered a genuine CCR, showed preferential expression in the xylem (Figure 3A), while both *MaCCR3/4* and *MaCCR6* were preferentially expressed in leaves (Figure 3B,C). These results further confirmed the possible functional differentiation of *MaCCRs* and suggested that *MaCCR1* may participate in the lignin biosynthesis in mulberry.

### 2.3. Enzymatic Assay of MaCCRs

**Figure 4:**
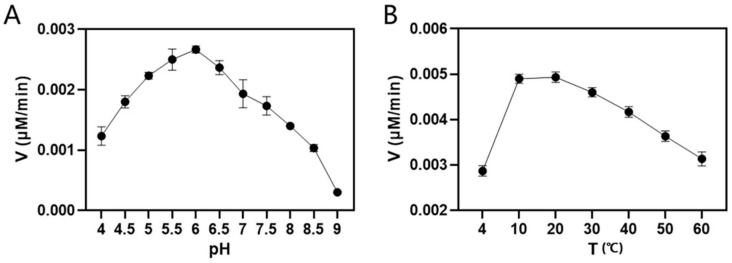
Optimum pH and temperature for *MaCCR1* with feruloyl CoA as substrate. (**A**) The activity of *MaCCR1* at different pH; (**B**) The activity of *MaCCR1* at different temperatures. Data are presented as means ± SD of three replicates.

Since *MaCCR3* and *MaCCR4* encode the same protein, MaCCR3 was used for enzymatic analysis and denoted as *MACCR3/4*. *MaCCR1*, *MaCCR3/4* and *MaCCR6* were successfully purified after prokaryotic expression (Figure S2). The concentration of purified *MaCCRs* ranged from 0.49 to 2.37 μg/μL. The enzymatic assay using feruloyl CoA and sinapyl CoA as substrates showed that *MaCCR1* can catalyze both substrates, while *MaCCR3/4* and *MaCCR6* showed no activity for the two substrates. Using feruloyl CoA as substrate, the optimal pH value and temperature for *MaCCR1* in vitro were identified as 6.0 and 20 °C, respectively (Figure 4). The kinetic analysis showed that *MaCCR1* had a lower Km value for feruloyl CoA and about three times higher *Kenz* value (*K_cat_/K_m_*) than for sinapyl CoA. Therefore, *MaCCR1* had affinity and higher catalytic efficiency for feruloyl CoA (Table 1).

### 2.4. The Lignin Content Was Changed by Knocking Down MaCCR1

**Figure 5:**
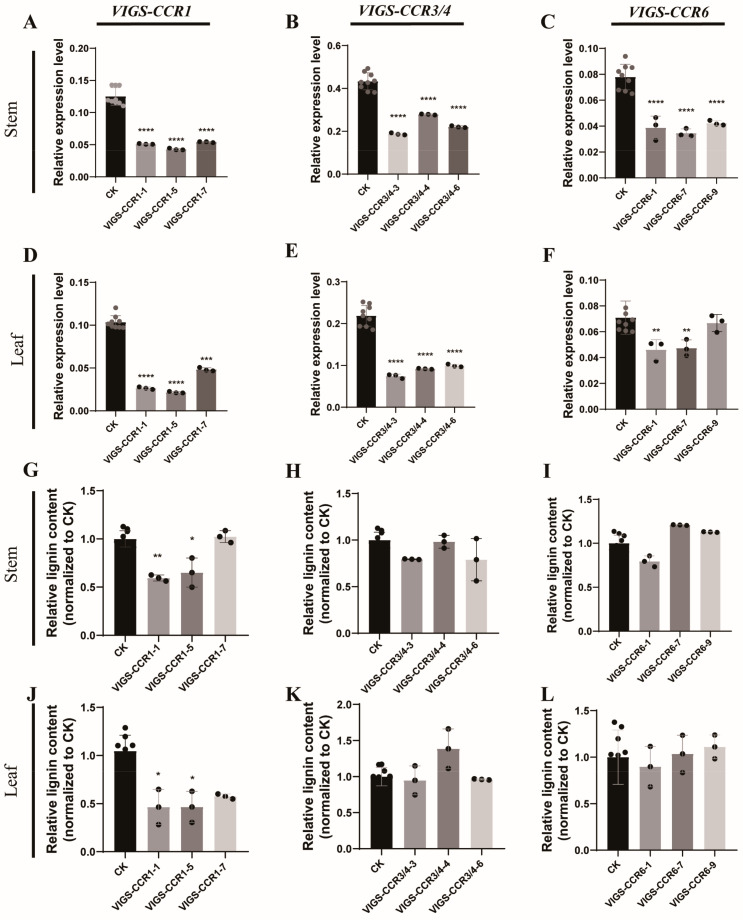
Knock-down of *MaCCRs* in mulberry stems and leaves and the corresponding lignin content. (**A**–**F**) Expression of *MaCCR1*, *MaCCR3/4* and *MaCCR6* in mulberry stem and leaf after VIGS treatment; (**G**–**L**) Lignin content in stems and leaves of mulberry trees after *MaCCR1*, *MaCCR3/4* and *MaCCR6* were knocked down. The lignin contents were measured and normalized to CK. Mulberry plants treated with TRV2 and TRV1 empty vectors were used as controls. VIGS lines for *MaCCR1*: VIGS-CCR1-1, VIGS-CCR1-5 and VIGS-CCR1-7; VIGS lines for *MaCCR3/4*: VIGS-CCR3/4-3, VIGS-CCR3/4-4 and VIGS-CCR3/4-6; VIGS lines for *MaCCR6*: VIGS-CCR6-1, VIGS-CCR6-7 and VIGS-CCR9-7. Data are presented as means ± SD. One-way ANOVA and Tukey’s multiple comparisons test were performed. Significant analysis with * (0.01 < *p* < 0.05), ** (0.005 < *p* < 0.01), *** (0.0001 < *p* < 0.005), **** (*p* < 0.0001).

**Figure 6:**
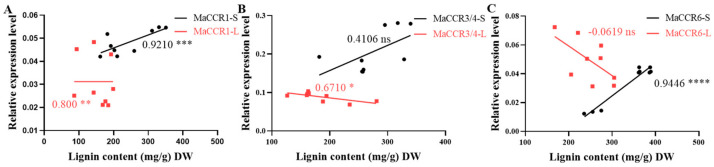
Correlation of *MaCCR* expression levels with lignin contents in mulberry. (**A**−**C**) The red line indicates the correlation between lignin content and *MaCCR1*, *MaCCR3/4* and *MaCCR6* gene expression level in leaves. Biological repeats are represented by red squares. The black line indicates the correlation between lignin content and *MaCCR1*, *MaCCR3/4* and *MaCCR6* gene expression level in stems. Biological repeats are represented by black squares. Significant analysis with * (0.01 < *p* < 0.05), ** (0.005 < *p* < 0.01), *** (0.0001 < *p* < 0.005), **** (*p* < 0.0001).

*MaCCRs* were knocked down using VIGS. Homologous segments were selected for simultaneous knock-down of *MaCCR3* and *4*. The knock-down degrees of *MaCCRs* in the treated mulberry seedlings were detected by qRT-PCR (Figure 5A–F). *MaCCR1*, *MaCCR3/4* and *MaCCR6* could be significantly down-regulated in both stems and leaves. However, the down-regulation of different *MaCCR* homologs in mulberry resulted in quite different effects on lignin contents. Significant down-regulation of *MaCCR3/4* (43.0–63.9% decrease in expression levels in different VIGS lines) or *MaCCR6* (45.7–55.5% decrease in expression levels in different VIGS lines) led to only a slight decrease or even slight increase in lignin content (Figure 5H,I,K,L). Significant down-regulation of the expression level of *MaCCR1 (VIGS-CCR1-1# and VIGS-CCR1-4#)* in stems and leaves resulted in a significant reduction in lignin content but kept the same growth condition as controls (Figure 5G,J, Table 2 and Figure S4). The correlation analysis showed that a significant positive correlation between the expression level of *MaCCR1* and the lignin content in different organs was observed (Figure 6). These results revealed that *MaCCR1* should be the dominant *CCR* involved in lignin biosynthesis in mulberry and could affect lignin content in both stem and leaf.

### 2.5. Simultaneous Down-Regulation of MaCCR1 and MaCAD3/4 Dramatically Decreased Lignin Content and Resulted in Growth Retardant in Mulberry

**Figure 7:**
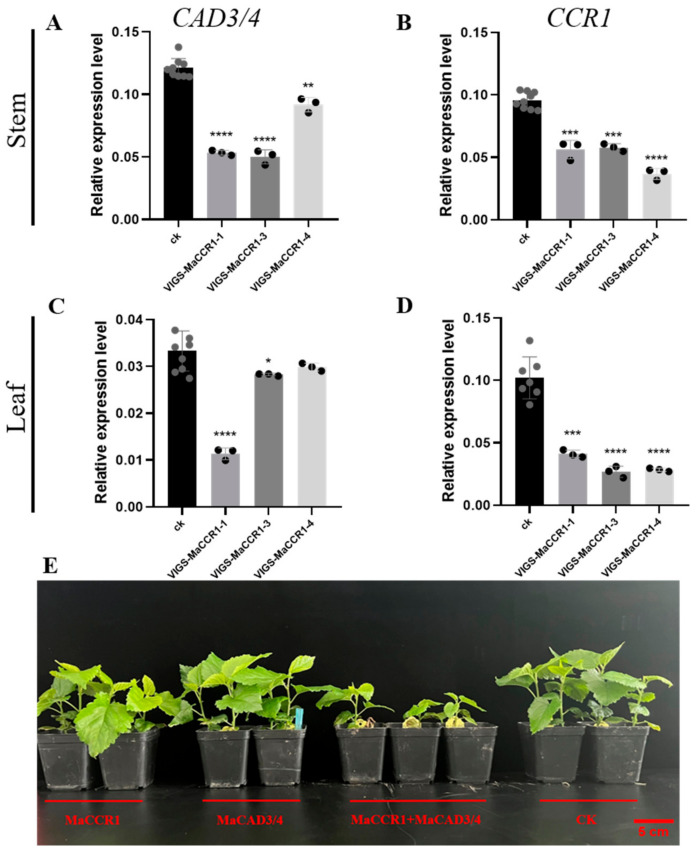
Knock-down of *MaCAD3/4* and *MaCCR1* in mulberry stems and leaves. (**A**–**D**) Expression of *MaCAD3/4* and *MaCCR1* in mulberry stem and leaf after treatment. (**E**) Mulberry phenotypes of *VIGS-MaCCR1*, *VIGS-MaCAD3/4*, *VIGS-MaCCR1+MaCAD3/4* and CK lines. Plants were incubated for 16 h/8 h in an artificial climate incubator. The scale bar indicating 5 cm is shown as a red line; data are presented as means ± SD of three biological replicates. One-way ANOVA and Tukey’s multiple comparisons test were performed. * (0.01 < *p* < 0.05), ** (0.005 < *p* < 0.01), *** (0.0001 < *p* < 0.005), **** (*p* < 0.0001).

*MaCCR1* and *MaCAD3/4* are the validated dominant genes involved in lignin biosynthesis based on previous studies and this study. The simultaneous knock-down degrees of *MaCCR1* and *MaCAD3/4* in the treated mulberry seedlings were detected by qRT-PCR (Figure 7A–D). It is obvious that significant down-regulation of *MaCCR1* and *MaCAD3/4* in stems and leaves at the same time can lead to a significant decrease in lignin content in mulberry (Figure 7A–D and Table 2). In addition, the plant growth and total biomass were also greatly affected. Compared with the control group, down-regulation of *MaCCR1* alone, which resulted in a decrease in lignin content, had almost no effect on plant height and total biomass (Figure 7E and Table 2). Down-regulation of *MaCAD3/4* alone can reduce lignin content but have no adverse effects on plant growth, with the plant height increasing by 4.3% and total biomass increasing by 83% (Figure 7E and Table 2). However, when *MaCAD3/4* and *MaCCR1* were down-regulated at the same time, a significant decrease in lignin content and dwarfing of the treated plants with reduced biomass were observed (Table 2 and Figure S1). 

## 3. Discussion

### 3.1. Dominant Genes in the Last Two Reductive Steps in Monolignol Biosynthesis in Mulberry

To date, a total of 11 enzymes have been identified as participants in monolignol biosynthesis, with the majority being encoded by multi-gene families in plants [2,12]. The CCR and CAD enzymes catalyze successive reductive steps and play crucial roles in determining both the content and composition of lignin [41]. Gene families encoding CCR and CAD have been documented across numerous plant species. Several studies have highlighted functional divergence and instances of mis-annotation within CCR and CAD gene families. The characterization of predominant CCR and CAD enzymes, along with the identification of target homologs, is fundamental for the genetic modification of lignin content and composition. Furthermore, the compensation of CCR and CAD homologs in mutants is considered to significantly influence the final phenotype [21,25,42]. In P. tomentosa, there are 11 genes encoding CCR or CCR-like proteins; however, only *PtoCCR1* and *PtoCCR7* have been identified as authentic *CCRs* involved in monolignol biosynthesis [37]. In M. truncatula, *MtCCR1* and *MtCCR2* have been characterized as genuine *CCRs*, exhibiting distinct substrate preferences. Mutations in *MtCCR1* and *MtCCR2* result in markedly different effects on lignin content, highlighting *MtCCR1* as the predominant *CCR* involved in monolignol biosynthesis [21]. The sequence divergence and conservation of CCRs in plants have been extensively studied, with signature motifs and specific binding motifs (SBM) identified for authentic CCRs, providing a foundational basis for distinguishing CCRs from CCR-like sequences [40]. In *Arabidopsis*, mis-annotated CAD genes have been reported, prompting a reclassification of AtCAD homologs [27]. Ultimately, *AtCAD4* and *AtCAD5* were characterized as the predominant *CADs* through comprehensive *in vitro* and *in vivo* functional analyses [27]. Genome-wide analyses of the *CCR* and *CAD* gene families have identified CCR1 and CAD3/4 as the key genes involved in lignin biosynthesis in *Morus notabilis* [2]. Our recent investigation into the *CAD* gene family in mulberry substantiates the role of *MaCAD3/4* as the predominant *CAD* enzymes in lignin biosynthesis. In the current study, *MaCCR1* was established as the principal CCR enzyme for lignin biosynthesis, following a systematic functional evaluation of MaCCR homologs in mulberry. *MaCCR1* is classified within the authentic CCR clade in phylogenetic analyses and possesses a conserved substrate-binding motif (SBM), thereby offering an additional example of SBM-based differentiation between CCR and CCR-like enzymes. Furthermore, *MaCCR1* exhibited elevated activity toward feruloyl CoA, consistent with the characteristics of most documented genuine CCRs in angiosperms [37]. The expression profile and enzymatic assay further confirmed *MaCCR1* as the authentic *CCR*. Silencing *MaCCR1* led to a reduction in lignin content, whereas the down-regulation of other *MaCCR* homologs did not result in a decrease in lignin content. Currently, *MaCCR1* and *MaCAD3/4* are clearly identified as the predominant genes responsible for lignin accumulation in mulberry.

### 3.2. Simultaneous Down-Regulation of MaCCR1 and MaCAD3/4 Induced Dwarfing Phenotype in Mulberry and Dramatic Decrease in Lignin Content

The extent of target gene down-regulation affects the phenotype, with *VIGS-MaCCR1-7#* showing a moderate lignin decrease, while *VIGS-MaCCR1-1#* and *VIGS-MaCCR1-5#* exhibited significant reductions. All MaCCR1-suppressed mulberries grew normally. A strong positive correlation between *MaCCR1* expression and lignin content was found, similar to observations in other plants like poplar when their main *CCR* genes are down-regulated [19]. The down-regulation of *MaCAD3/4* led to a significant reduction in lignin content while concurrently enhancing biomass production. This phenomenon of increased biomass as a result of *CAD* gene down-regulation has also been observed in CAD-RNAi maize [43]. Only significant down-regulation or mutations of dominant plant CADs typically lead to reddish stem or xylem phenotypes. VIGS is an unstable genetic modification with varying down-regulation levels in each experiment. In this study, down-regulating *MaCAD3/4* did not change stem color, whereas our previous study showed that a 70% down-regulation caused a brownish-red stem phenotype [34]. The natural *cad* mutant mulberry (*Morus alba*), known as ‘Sekizaisou’, was identified by its red-colored wood [44]. In numerous species, *CCR* and *CAD* genes demonstrate similar expression patterns within vascular tissues. Specifically, the spatial co-expression of *MaCCR1* and *MaCAD3/4* was observed, with a preferential expression in stems or xylem [34]. Furthermore, RNA interference (RNAi) suppression of *PtrCAD1* in *Populus trichocarpa* resulted in a decrease in CCR activity within stem-differentiating xylem (SDX). Additionally, physical interactions between PtrCAD1 and PtrCCR2 were confirmed, forming a heterodimer that influences their activity in monolignol biosynthesis [45]. Consequently, efforts have been made to concurrently regulate the predominant *CCR* and *CAD* genes to alter lignin content. Notably, a significant reduction in lignin content has been observed through the simultaneous down-regulation of *CCR* and *CAD* in both tobacco and *Arabidopsis* [27,28]. In our investigation, the concurrent down-regulation of *MaCCR1* and *MaCAD3/4* resulted in a pronounced decrease in lignin content, accompanied by a dwarf phenotype and reduced biomass in mulberry. A similar severe dwarf phenotype was also observed in *Arabidopsis* following the simultaneous suppression of *CCR* and *CAD* [46].

## 4. Materials and Methods

### 4.1. Plant Materials

The roots, stems and leaves of 1-year-old *Morus alba var. Fengchi* growing in Zhenjiang, Jiangsu Province (north latitude 32°11′, east 119°27′), were collected. Xylem was scraped from the exposed wood after removal of the bark, and the phloem was removed and collected by scraping the bark from which the cork was removed [47,48]. Virus-induced gene silencing (VIGS) was performed on 3-week-old seedlings of *Morus alba var. Fengchi.* Plants were grown in a growth chamber at 22 °C with a 16/8 day/night cycle and 40–60% humidity. The collected samples were frozen with liquid nitrogen immediately and stored at −80 °C until use. At least three biological replicates were collected.

### 4.2. RNA Extraction and cDNA Synthesis

RNA was extracted from different mulberry tissues (roots, stems, leaves, xylem and phloem) according to the instructions contained in the EASYspin Plus Polysaccharide Polyphenol Complex Plant RNA Rapid Extraction Kit (Aidlab, Beijing, China), and RNA quality was detected via agarose gel electrophoresis and NanoDrop One (Thermo Scientific, Waltham, MA, USA). cDNA synthesis was performed using R323-01 HiScript III RT kit (Vazyme, Nanjing, China) according to its manufacturer’s protocol.

### 4.3. Cloning of CCR Genes in Mulberry

Specific primers for cloning *CCR* genes were designed based on the sequence information (L484_013511.p01,L484_013509.p01,L484_015311.p01,L484_015312.p01,L484_015313.p01 and L484_015314.p01) of functional genes screened from the mulberry genome (https://morus.biodb.org/ accessed on 20 March 2021). *CCR* genes of mulberry (*Morus alba*) were cloned via three-step PCR at a gradient annealing temperature of 48~62 °C. The PCR products were purified using SanPrep column DNA gel recovery kit (Sangon Biotech, Shanghai, China) and cloned into TA/Blunt-Zero vector (Vazyme, Nanjing, China). The PCR products were amplified in *Escherichia coli DH5α* and confirmed by Sanger sequencing. Sequences were acquired using an ABI 3730xl Sequencer (ABI Applied Biosystems, Waltham, MA, USA) and analyzed using SnapGene version 3.1.1. The validated sequences were deposited in GenBank with accession numbers OQ378328, OQ378329, OQ378330 and OQ378331.

### 4.4. Phylogenetic Analysis and Sequence Alignment of CCRs

DNAman 9.0 (Lynnon BioSoft, https://www.lynnon.com/dnaman.html accessed on 15 May 2022) and MEGA 7.1 were used to align the obtained MaCCR with the CCR or CCR-like protein sequences from other plant species. MEGA 7.1 was used to infer the phylogenetic tree using these protein sequences; the maximum likelihood method with default settings was used; and a bootstrap with 1000 replications was carried out to evaluate the credibility of each branch.

### 4.5. Expression Profile of MaCCRs in Mulberry

Quantitative real-time PCR (qRT-PCR) was performed using the ABI StepOnePlus real-time PCR system (USA) to investigate the expression of *MaCCRs* in different tissues of mulberry. Actin was used as the reference gene. GraphPad Prism 8.0 was used for visualizing the results. *T*-test and ANOVA were performed, and *p* < 0.05 was considered significant. qRT-PCR was performed in three technical replicates with three biological replicates. The primers used in this study are available in Table S2.

### 4.6. Prokaryotic Expression and Purification of His-Tagged MaCCRs

The recombinant plasmids pET-28a-MaCCRs were constructed by seamless cloning with linearized pET-28a vector; thereafter, the recombinant plasmids were transformed into *DH5α* and confirmed by sequencing. The confirmed recombinant plasmids were transformed into *E. coli BL21* (DE3) and were then incubated in LB liquid medium containing kanamycin (50 μg/mL) at 37 °C until OD600 = 0.6. Isopropyl β-d-thiogalactosides (IPTG) were added at a final concentration of 0.6 mmol/L to induce the overexpression of the target protein. The cultures were incubated overnight at 16 °C under shaking at 220 rpm. Purification of His-tagged *MaCCRs* was performed according to a previous report (Chao et al. 2021a [37]). The quality of the eluted proteins was determined by SDS-PAGE and Coomassie brilliant blue G250 (Sangon Biotech, Shanghai, China).

### 4.7. Enzymatic Assay of MaCCRs

Using feruloyl CoA and sinapyl-CoA (ZZstandard, Shanghai, China) as substrates, the enzymatic kinetics were determined by detecting the decrease in the substrates based on absorbance. The detailed method was described in our previous studies [37,48]. Substrate reduction was measured using BioTek ^®^ Epoch 2 Microplate Reader (BioTek ^®^Epoch 2, BioTek Instruments, Inc., Winooski, VT, USA). The *K_m_* and *V_max_* values were determined via non-linear fitting and double reciprocal in GraphPad prism 8.0.

### 4.8. Virus-Induced Gene Silencing (VIGS) in Mulberry

The VIGS fragments of the target genes were cloned, and the recombinant plasmids pTRV2-MaCCRs and pTRV2-MaCAD3/4 were constructed by nimble cloning [49]. The recombinant plasmids pTRV2-MaCCRs, pTRV2-MaCAD3/4, pTRV2 and pTRV1 were transformed into Agrobacterium GV3101 using a freeze–thaw method [50]. Experiments were performed according to previously published methods in our laboratory [51]. The knock-down efficiency was determined by qRT-PCR. After infection, the plants were cultured in an artificial climate incubator for 16 h/8 h. Samples were collected after 4 weeks, frozen in liquid nitrogen.

### 4.9. Determination of Biomass and Lignin Content

Biomass was represented by plant fresh weight, and plant weights were recorded before sample collection [52]. The values of the mean plus standard deviation of all biological replicates were shown. The lignin content in the samples was determined using the lignin content assay kit (Sangon Biotech, Shanghai, China) according to the acetylation method described in the instructions. Lignin was stained using phloroglucinol according to the manual provided by Yuanye Bio-Technology (Shanghai, China), and the slices were observed and visualized under a microscope (Olympus BX53, Melville, NY, USA).

### 4.10. Statistical Analysis

Statistical tests were carried out in SPSS 19 (IBM, Armonk, NY, USA). Before statistical analysis, all data were checked for normality. One-way ANOVA and Tukey’s multiple comparisons test were performed. *p* < 0.05 marked a significant result.

## 5. Conclusions

In summary, we cloned and analyzed four CCR homologs in mulberry, identifying *MaCCR1* as the primary *CCR* through enzymatic and functional studies. Although *MaCCR1* expression correlated with lignin content, reducing the CCR slightly lowered lignin levels. However, jointly down-regulating *MaCCR1* and *MaCAD3/4* significantly reduced lignin but also led to reduced biomass and abnormal growth. Down-regulating *CAD3/4* alone significantly reduced lignin content while maintaining normal growth and increased biomass, suggesting *MaCAD3/4* as a promising target for lignin modification in mulberry. Our findings shed light on the impact of key *CCR* and *CAD* genes on lignin content in mulberry.

## Figures and Tables

**Table 1:** Enzymatic kinetic parameters of MaCCR1.

	Substrate	*K_m_* _(_μM)	*V_m_*_ax_ (μM min^−1^)	*K_cat_* (min^−1^)	*K_cat_/K_m_* (min^−1^ μM^−1^)
MaCCR1	feruloyl CoA	62.56	81.97	53.30	0.65
	sinapyl-CoA	160.48	49.02	31.88	0.20

**Table 2:** Plant growth after different VIGS treatments.

	Plant Height (cm)	Leaf Number	Leaf Spacing (cm)	Biomass (g)	Lignin (g/g DW)	
					Stem	Leaf
CK	6.78 ± 1.42	8 ± 1	3.31 ± 0.91	2.18 ± 0.95	0.32 ± 0.02	0.31 ± 0.02
VIGS-MaCCR1 + MaCAD3/4	3.58 ± 1.78 **	7 ± 1	1.63 ± 0.93 **	0.72 ± 0.51 **	0.26 ± 0.009 **	0.18 ± 0.03 *
VIGS-MaCAD3/4	7.07 ± 1.32	9 ± 1	3.28 ± 0.98	3.99 ± 0.89 **	0.34 ± 0.08 **	0.27 ± 0.06 **
VIGS-MaCCR1	6.79 ± 1.91	8 ± 1	2.99 ± 0.91	2.18 ± 1.22	0.24 ± 0.04 *	0.15 ± 0.05 *

Mean ± SD was used to show the results; Significant analysis with * (0.05 < *p* < 0. 01), ** (*p* < 0.01). At least three controls and VIGS lines for each group, respectively, were measured. CK: mulberry plants treated with empty TRV1 and TRV2.

## Data Availability

Data is contained within the article or Supplementary Materials.

## Supplementary Materials

The following supporting information can be downloaded at: https://www.mdpi.com/article/10.3390/plants13243512/s1, Table S1. Basic information on MaCCRs. Table S2. Primers used in this study. Table S3. Putative CCR protein sequences used for phylogenetic analysis. Figure S1. Plant height and biomass of mulberry after *MaCAD3/4* and *MaCCR1* knock-down. Figure S2. SDS-PAGE results for MaCCRs. Figure S3. VIGS vector map information. Figure S4. Phloroglucinol staining of lignin in VIGS down-regulation of *MaCCR1* transgenic mulberry.
