# Supplementary figures and images for: Simultaneous Down-Regulation of Dominant Cinnamoyl CoA Reductase and Cinnamyl Alcohol Dehydrogenase Dramatically Altered Lignin Content in Mulberry
Source: Plants (Basel). 2024 Dec 16;13(24):3512. doi: 10.3390/plants13243512 (PMC11676671; doi:10.3390/plants13243512)

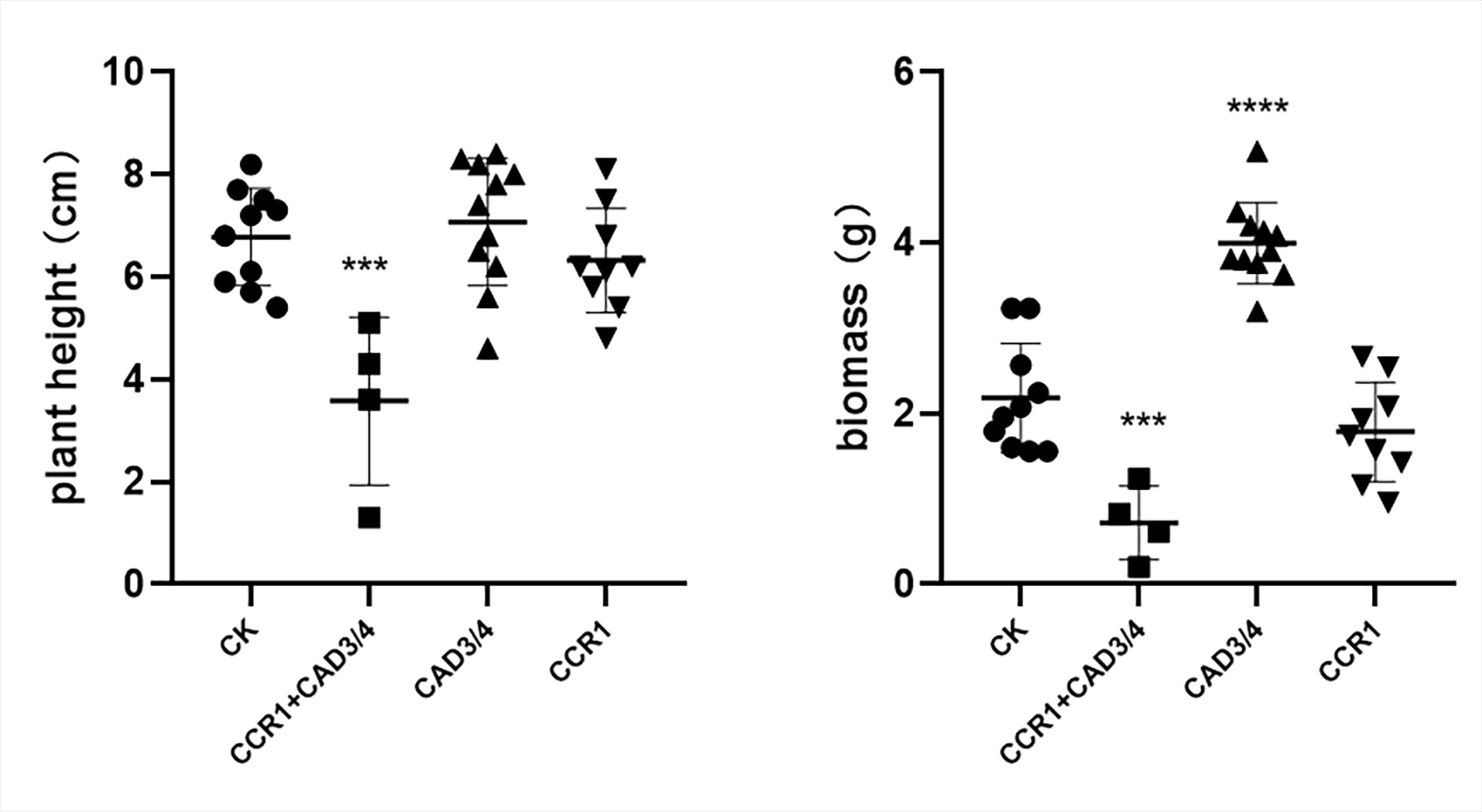

Supplement: Supplementary file 1 [file plants-13-03512-s001.zip › Figure S1.tif]

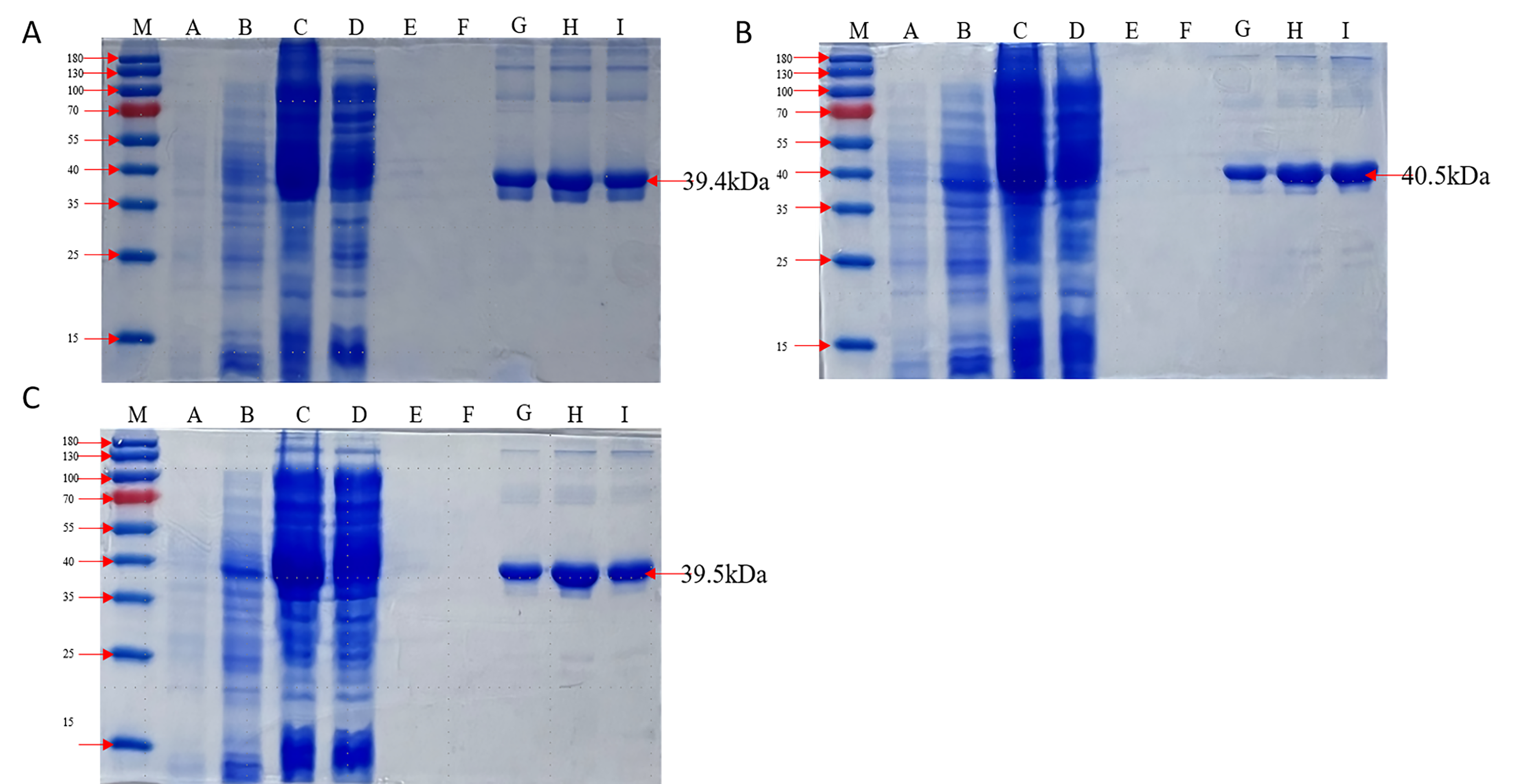

Supplement: Supplementary file 1 [file plants-13-03512-s001.zip › Figure S2.tif]

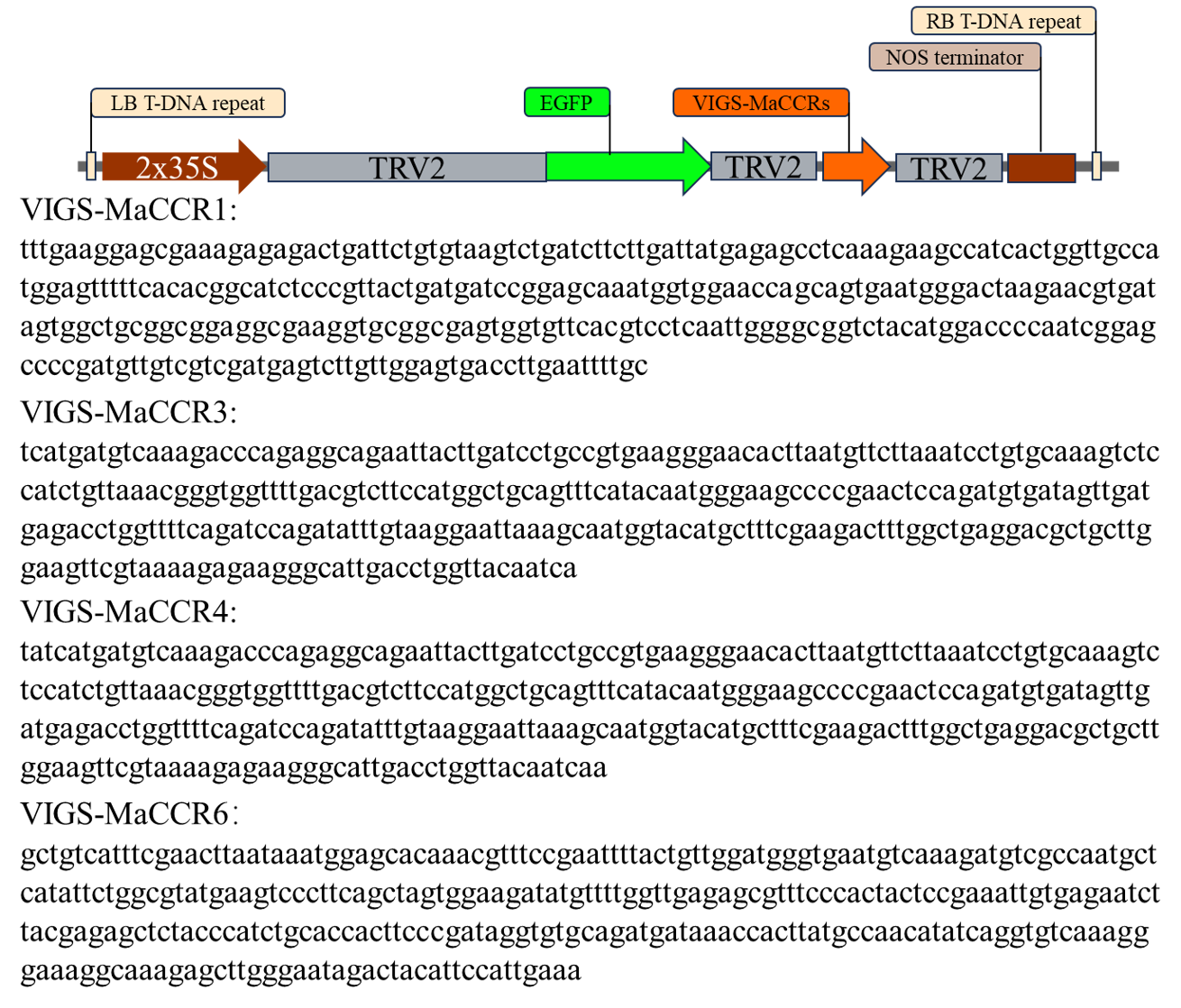

Supplement: Supplementary file 1 [file plants-13-03512-s001.zip › Figure S3.tif]

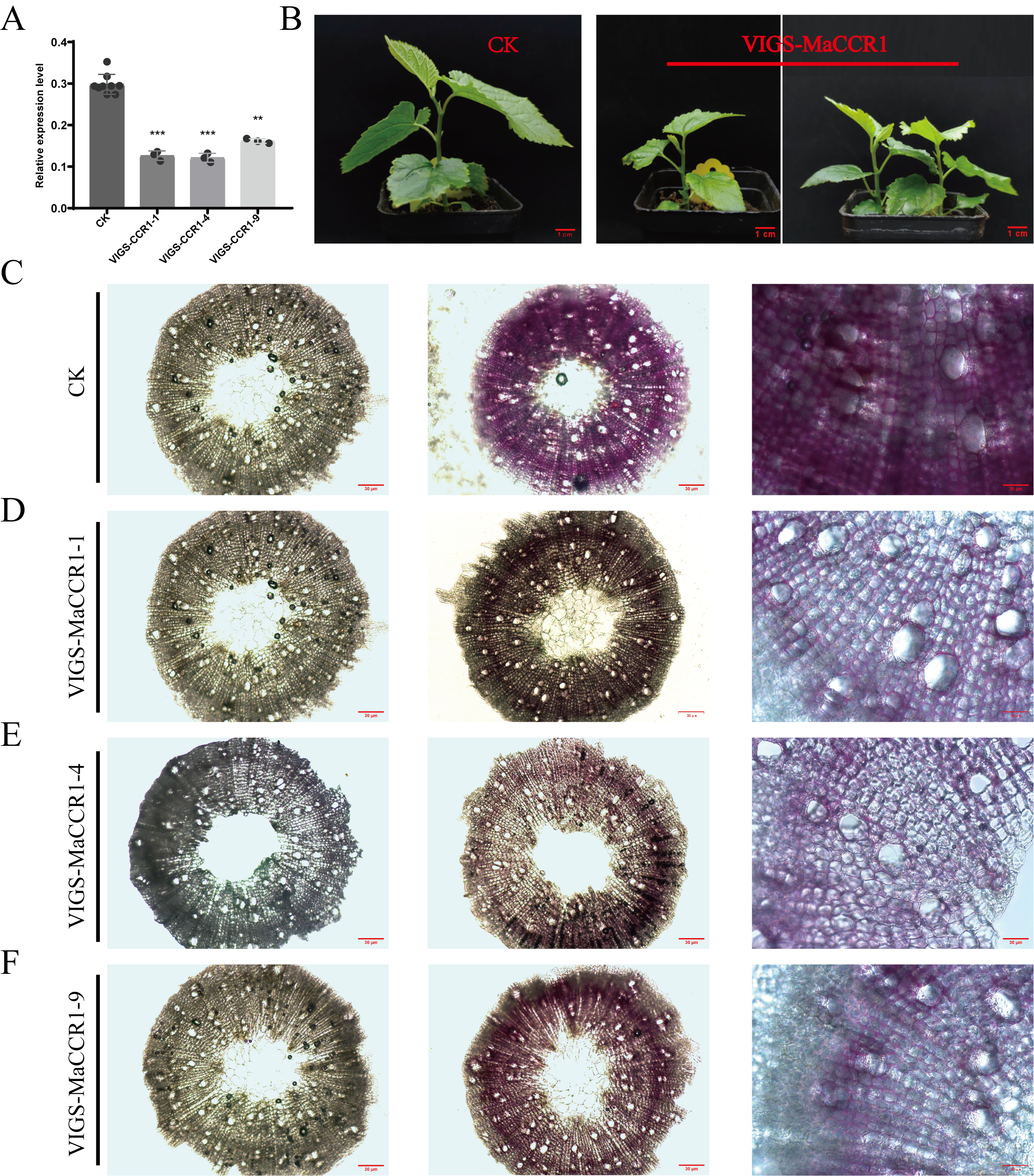

Supplement: Supplementary file 1 [file plants-13-03512-s001.zip › Figure S4.tif]
